# Recurrent neonatal sepsis and progressive white matter injury in a premature newborn culture-positive for group B *Streptococcus*: A case report

**DOI:** 10.1097/MD.0000000000026387

**Published:** 2021-06-25

**Authors:** Cheong-Jun Moon, Tae Hee Kwon, Kyung Sang Lee, Hyun-Seung Lee

**Affiliations:** a Department of Pediatrics, St. Vincent's Hospital, College of Medicine, The Catholic University of Korea; b Department of Radiology, Cha Gangnam Medical Center, Cha University School of Medicine; c Department of Pediatrics, Incheon Worker‘s Compensation Hospital, Incheon; d Department of Pediatrics, Cha Gangnam Medical Center, Cha University School of Medicine, Seoul, Republic of Korea.

**Keywords:** group B *Streptococcus*, neonatal sepsis, periventricular leukomalacia, recurrence, ventriculomegaly

## Abstract

**Rationale:**

Group B *Streptococcus* (GBS) remains a principal pathogen causing neonatal sepsis and meningitis, particularly in premature infants with relatively insufficient immunity. Recurrence may occur uncommonly, largely associated with subclinical mucosal persistence or repetitive exposure to exogenous sources. White matter injury (WMI) including cystic periventricular leukomalacia (PVL) has been associated with intrauterine infection/inflammation, and neonatal infection as a more significant predictor including postnatal sepsis and recurrent infection, even without microbial neuroinvasion. Furthermore, clinical and experimental evidence of WMI by some bacteria other than GBS without central nervous system invasion has been reported. However, there is little evidence of WMI associated with neonatal GBS sepsis in the absence of meningitis in the literature.

**Patient concerns:**

A newborn at 30^+4^ weeks’ gestation with low birthweight presented with 2 episodes (with a 13-day interval with no antibiotic therapy) of neonatal sepsis culture-proven for GBS with early-onset presentation after clinical chorioamnionitis via vertical GBS transmission and the associated conditions including prematurity-related neonatal immunodeficiency and persistent mucosal GBS carriage after the first antibiotic treatment. The perinatal GBS infection was complicated by progressive WMI presenting with ventriculomegaly and cystic PVL without a definite evidence of meningitis, intraventricular hemorrhage, and documented cerebral hypoxia or hypoperfusion conditions including septic shock.

**Diagnoses:**

Recurrent group B streptococcal sepsis and cystic PVL with ventriculomegaly.

**Interventions:**

Two episodes of GBS sepsis were treated with 15-day parenteral antibiotic therapy, respectively.

**Outcomes:**

Resolution of the recurrent GBS sepsis without further relapses, however, complicated by WMI and subsequent about 6 months delay in motor development at 12 months’ corrected age.

**Lessons:**

This case suggests WMI associated with GBS bacteremia without central nervous system entry by viable GBS and also shows that in premature infants, intrauterine GBS infection with no interventions may lead to extensive and persistent GBS colonization, early-onset and recurrent GBS disease, and WMI. Postnatal as well as intrauterine infection/inflammation controls with maternal prophylaxis may be pivotal for prevention and limiting the magnitude of neurologic injury.

## 1 Introduction

Group B *Streptococcus* (GBS) or *Streptococcus agalactiae* remains a principal pathogen causing sepsis and meningitis in young infants.^[1]^ This Gram-positive microorganism is a resident flora of the alimentary and urogenital tracts. Neonatal GBS disease is classified into early-onset disease (occurring 0–6 days of life) and late-onset disease (7–89 days).^[2]^ Early-onset disease may result from mother-to-child transmission via ascending intrauterine infection through ruptured or intact fetal membranes^[3]^ and/or intrapartum direct inoculation by contacting with maternal blood and vaginal secretions.^[1]^ Intrapartum chemoprophylaxis has been shown a beneficial effect on early-onset disease, but not late-onset disease.^[4]^ Postnatal exogenous acquisition from nursery personnel, household, and community or nosocomial sources has been implicated in late-onset disease.^[5]^ Recurrence may occur in 0.5% to 4.5% of infected infants,^[6]^ frequently preterm babies, due to original or unrelated strains via the following estimated mechanisms: subclinical persistent mucosal colonization related to various factors including insufficient host immunity, inadequate treatment dosage or duration, and microbial hypervirulence and resistance and repetitive exposure to the exogenous sources including GBS-contaminated breast milk which is linked to high recurrence rates and severe presentation.^[7,8]^

Cerebral white matter injury (WMI) includes focal necrosis (severe form) causing preoligodendrocyte death that evolves into macroscopic cysts (grade II–IV periventricular leukomalacia [PVL]) or microcysts and diffuse non-necrotic lesions (predominant form) leading to aberrant preoligodendrocyte maturation, both resulting in myelination disturbance.^[9]^ Premature infants may be at the greatest risk for WMI associated with a developmental window of 23 to 32 gestational weeks characterized by immature white matter features of poor vascularization with impaired cerebral flow autoregulation and maturation-dependent vulnerability of oligodendrocyte progenitors to various insults.^[9,10]^ Diffuse WMI leading to white matter paucity can be accompanied by ventriculomegaly (defined as lateral ventricle widths ≥10 mm) without increased intraventricular pressure.^[11,12]^ WMI has been associated with intrauterine infection/inflammation and postnatal infection including sepsis^[13–16]^ and recurrent infection.^[17]^ Several studies of preterm infants demonstrated that postnatal infection that can further augment the inflammatory response associated with brain injury is a more crucial associated factor for WMI, particularly nonhemorrhagic cystic PVL,^[18]^ than chorioamnionitis.^[19–21]^ Clinical and experimental evidence that bacteremias including *Escherichia coli* bacteremia and *Staphylococcus epidermidis* bacteremia elicit WMI without penetration of viable bacteria into the central nervous system (CNS) has been found.^[13,14,16,17]^ However, there is little evidence of WMI associated with neonatal GBS sepsis without meningitis, which suggests a causal relationship between GBS sepsis without CNS invasion and neonatal brain injury, in the literature.

Herein, we report a rare case of 2 episodes of neonatal sepsis culture-proven for GBS with early-onset presentation after clinical chorioamnionitis via vertical GBS transmission in a premature newborn with prematurity-related neonatal immunodeficiency and persistent mucosal GBS carriage after the first antibiotic treatment. The perinatal GBS infection was complicated by progressive WMI presenting with nonhemorrhagic ventriculomegaly and cystic PVL without a definite evidence of meningitis and documented cerebral hypoxia or hypoperfusion conditions including septic shock.

## 2 Ethics approval

Written informed consent was obtained from the patient's legal guardian for the anonymized clinical data including accompanying images to be analyzed and published for research purposes. The present study was approved by the Institutional Review Board (IRB) of CHA Gangnam Medical Center (IRB No. GCI 2021–02–002).

## 3 Case report

**Figure 1:**
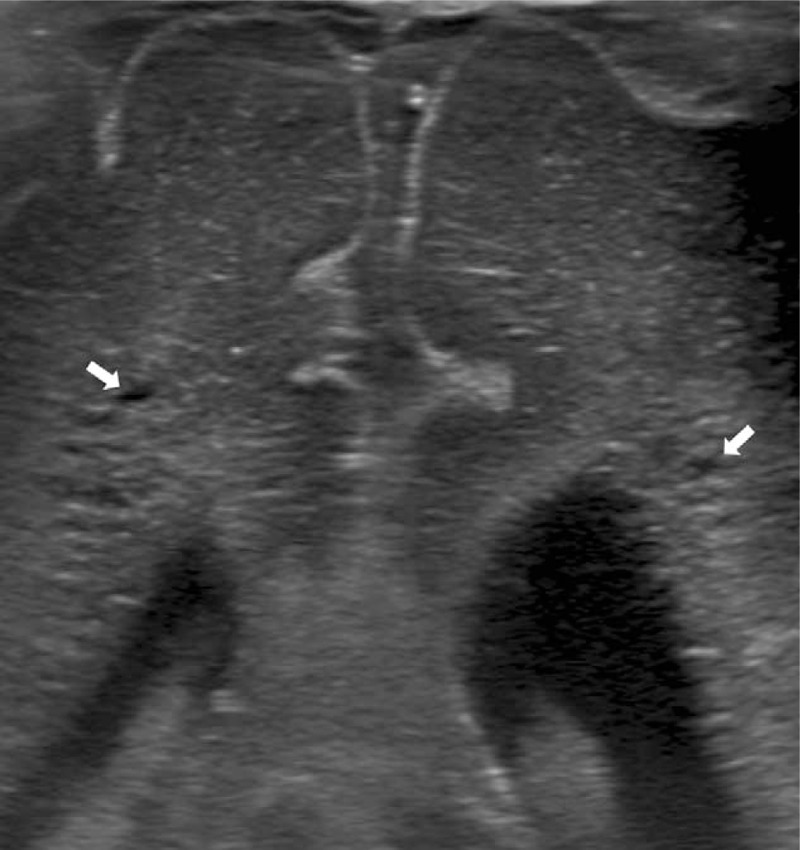
Coronal, magnified view of gray scale cranial sonogram at the level the atria of the lateral ventricles on postnatal day 16 demonstrates white matter damage composed of bilateral increased periventricular echogenecities with small cystic degenerations (white arrows).

**Figure 2:**
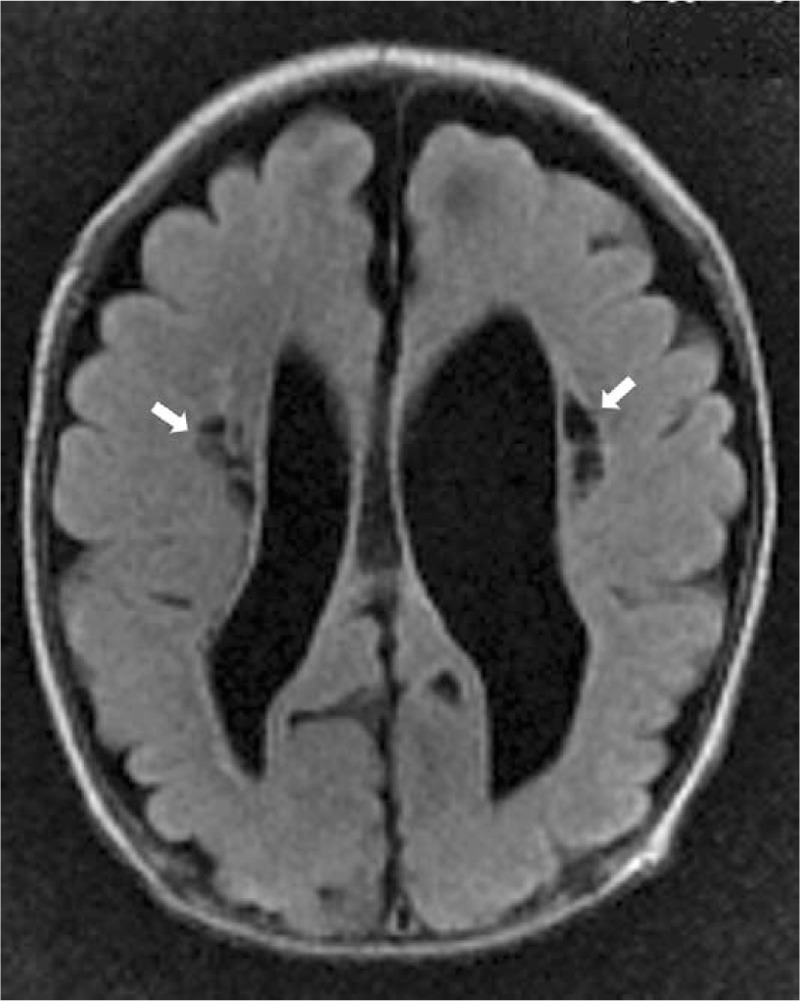
Axial T2-weighted FLAIR magnetic resonance imaging scan at the mid-ventricular level on postnatal day 51 (at 37^+5^ weeks’ postmenstrual age) depicts cystic periventricular leukomalacia, appearing as circumscribed areas of linear and focal low signal intensities (white arrows) within the cerebral white matter abutting the external angles of the lateral ventricles, and asymmetric enlargement of the lateral ventricles (more prominent on the left). FLAIR = fluid-attenuated inversion recovery.

A Korean male infant with a weight of 1760 g (81st percentile) was born to a 22-year-old primigravid woman at 30^+4^ weeks’ gestation via an emergency cesarean section delivery due to fetal distress with 2-day duration of premature rupture of the membranes (PROM). The mother was diagnosed with clinical chorioamnionitis based on the clinical findings at admission: a body temperature of 38.4 °C, a total white blood cell (WBC) count of 23,220 cells/mm^3^ with 89% polymorphonuclear leukocytes, a heart rate of 129 beats/min, uterine fundal tenderness, and foul odor of the amniotic fluid. The mother had received no antenatal care including rectovaginal GBS screening and antepartum antibiotic administration without other particular medical history. Apgar scores were 4 and 7 at 1 and 5 minutes after birth, respectively. At birth, the infant presented with no initial crying, depressed muscle tone, and a heart rate of <100 beats/min, improved with resuscitation with positive pressure ventilation. Because of poor respiratory effort, the infant received ventilator support for 3 days with an episode of respiratory distress syndrome ameliorated by administrating a single dose of exogenous surfactant. The infant's body temperature was 36.9 °C. Empiric antimicrobial therapy with parenteral ampicillin (200 mg/kg/d) and gentamicin (4.5 mg/kg/36 h) was initiated for suspected perinatally acquired sepsis. The cord blood culture showed rapid growth of Gram-positive cocci in chains at 8 hours incubation, identified as *S agalactiae* at 48 hours. The mother developed postoperative surgical site infection with pelvic abscesses. Cultures from the maternal surgical wound and abscesses 2 days postpartum revealed GBS. Table 1 shows the flow of the infant's laboratory and clinical data. Initial cultures of the infant's blood, nasal mucosa, gastric aspirate, rectum and axilla specimens all grew GBS, and the urine culture was sterile. Initial laboratory evaluation demonstrated elevated C-reactive protein (CRP, 1.05 vs control <0.3 mg/dL). Blood culture repeated after 48 hours of antimicrobial administration was sterile. On postnatal day 5, gentamicin was discontinued. Ampicillin was ceased on day 15. On day 24, it was noted that the nasal mucosal swab culture performed 7 days after cessation of antibiotic administration yielded GBS. However, oral rifampicin as a decolonization therapy was not considered. On day 28 (13 days after completion of the first course of antibiotic therapy), respiratory distress was noted with apnea, a heart rate of 70 beats/min, and oxygen saturation 60% on room air. Cultures from blood and urine were obtained. The infant was afebrile (37.2 °C). The WBC count was 8140 cells/mm^3^ with 16% segmented neutrophils and 71% lymphocytes. The CRP level was initially negative (<0.03 mg/dL), however, 2 days later the positive conversion (3.93 mg/dL) was observed. Initial broad-spectrum antimicrobial therapy consisted of 5 days of intravenous vancomycin, ampicillin, and gentamicin. Subsequent to antimicrobial therapy, the symptoms promptly resolved. The blood culture yielded GBS at 48 hours incubation and ampicillin was sustained for a total duration of 15 days. The urine culture was sterile. After 48 hours of antibiotic administration, repeat blood culture was sterile and cerebrospinal fluid (CSF) analysis and culture showed a WBC count of 1 cell/mm^3^, a protein level of 150 mg/dL, a glucose level of 39 mg/dL, and CSF sterility. Serial sonographic brain scanning on days 2, 16, 31, and 47 revealed progressive WMI consisting of bilateral periventricular echodensities evolving into focal cystic degeneration (Fig. 1) and bilateral asymmetric lateral ventriculomegaly, which was confirmed by brain magnetic resonance imaging on day 51 (Fig. 2). Quantitative immunoglobulin profile was evaluated in the cord serum and the infant's serum on day 36; all the assessed levels of immunoglobulins G, A, and M were lower than the reference values established in similar-aged healthy infants. The Profile of T-, B-, and natural killer cell subset percentages on day 36 showed normal values. Complement profile was evaluated on day 42 of hemolytic complement activity, C3 and C4, of which all the serum levels were decreased compared with the reference values determined in sera of premature or term infants comparable in postnatal age. The infant's initial feeds comprised total parenteral nutrition, which maintained until day 13, and gavage feeds with preterm formula milk with no introduction to breastfeeding during the admission period. Oral feeding was added on day 14 and full oral feeding was achieved on day 37. Cultures from the infant's blood and nose and axilla swabs on days 36 and 49 were negative for GBS. Serological assays for toxoplasmosis, rubella, cytomegalovirus, herpes, and human immunodeficiency virus were negative. Abdominal ultrasound, echocardiography, and radiographic skeletal survey examination were normal. After the second set of GBS sepsis episodes, no further relapses have occurred. However, the infant was about 6 months delayed in motor development at 15 months of follow-up (12 months’ corrected age).

**Table 1:** Laboratory and clinical data of the present case.

Parameters	Day 1	Day 3	Day 15	Day 22	Day 28	Day 30	Day 36	Day 49
Postmenstrual age, wks	30+4	30+6	32+4	33+4	34+3	34+5	35+4	37+3
Hemoglobin, g/dL	17.3	13.8	11.5	13.1	10.9	9.0	8.7	10.3
Hematocrit (%)	49.7	38.8	32.6	36.5	30.8	25.6	25.7	30.3
Platelets, /mm^3^	326,000	309,000	495,000	447,000	385,000	284,000	427,000	270,000
WBC count, /mm^3^	6860	7710	14130	11560	8140	7480	9640	6140
Seg neutrophils (%)	35	49	34	21	16	28	21	15
Lymphocytes, /mm^3^	53	41	54	65	71	59	65	78
CRP, mg/dL	1.05	0.44	<0.03	<0.03	<0.03	3.93	0.23	<0.03
Ig G (700–1600 mg/dL)^∗^	437^†^	–	–	–	–	–	232^‡^	–
Ig A (70–400 mg/dL)^∗^	–	–	–	–	–	–	<6.6^‡^	–
Ig M (40–230 mg/dL)^∗^	<19.0^†^	–	–	–	–	–	19.2^‡^	–
CH50 (23–46 U/mL)^∗^	–	–	–	–	–	–	<5.0^§^ (day 42)	–
C3 (90–180 mg/dL)^∗^	–	–	–	–	–	–	38.2^§^ (day 42)	–
C4 (10–40 mg/dL)^∗^	–	–	–	–	–	–	<5.3^§^ (day 42)	–
CSF analysis	–	–	–	–	–	WBC 1 cell/mm^3^ Protein 150 mg/dL Glucose 39 mg/dL	–	–
GBS culture results	Blood (+) Nose, axilla, gastric, and rectal swabs (+) Urine (–) Intubation tip (–)	Blood (–)	PICC tip (–)	Nose swab (+) Axilla swab (–)	Blood (+) Urine (–)	Blood (–) CSF (–) Gastric and rectal swabs (–)	Blood (–) Nose and axilla swabs (–) Day 41 PICC tip (–)	Blood (–) Nose and axilla swabs (–)
Neuroimaging findings	Day 2 cranial sonography: bilateral PVE (early grade I PVL) and left lateral ventriculomegaly (10 mm-sized atrial diameter)		Day 16 cranial sonography: bilateral PVE with tiny cystic alterations (grade II PVL) and left lateral ventriculomegaly (11.6 mm), worse than the last study findings			Day 31 cranial sonography: bilateral PVE with cystic alterations (grade II PVL), left lateral ventriculomegaly (12.6 mm), and right lateral ventriculomegaly (10 mm), worse than the last study findings		Day 47 cranial sonography: bilateral PVE with cystic changes, left lateral ventriculomegaly (15 mm), and right lateral ventriculomegaly (12 mm), worse than the last study findings Day 51 brain MRI: WMI with cystic formation (grade II PVL) and bilateral asymmetric lateral ventriculomegaly
Clinical notes	1st sepsis episode Parenteral ampicillin for 15 days with 5 days of gentamicin		1st antibiotic therapy cessation	Interval between episodes	2nd sepsis episode Parenteral ampicillin for 15 days with 5 days of vancomycin and gentamicin			7 days after the 2nd antibiotic therapy cessation

CH50 = total hemolytic complement 50, CRP = C-reactive protein, CSF = cerebrospinal fluid, GBS = group B *Streptococcus*, Ig = immunoglobulin, PICC = peripherally inserted central catheter, PVE = periventricular echogenicity, PVL = periventricular leukomalacia, Seg neutrophils = segmented neutrophils, WBC = white blood cell, WMI = white matter injury.

∗ Reference values in adults.

† Data from the umbilical cord blood examination and reference values in similar-aged healthy infants: Ig G 636–1606 and Ig M 23–196 mg/dL.

‡ Reference ranges in similar-aged healthy infants: Ig G 251–906 mg/dL, Ig A 9–142 mg/dL, and Ig M 23–196 mg/dL.

§ Reference mean levels (± standard deviation): CH50 47.5 U/mL for preterm neonates; C3 97.5 (±17.4) mg/dL for term neonates; and C4 37.4 (± 9.9) mg/dL for term neonates.

## 4 Discussion

Our prematurely born infant showed 2 episodes of GBS sepsis without focal infections (initial early-onset and recurrent sepsis) with a 13-day interval between the first therapy course completion and the second episode onset, both of which were treated with 15 days of antibiotic therapy, respectively. However, progressive WMI was complicated.

The infant had previously reported risk factors for early-onset GBS disease including PROM and clinical chorioamnionitis with intrapartum fever as obstetric factors and prematurity and low birthweight as host factors. The culture results from maternal wound sites and abscesses, cord blood, and infant's sites evidenced vertical GBS transmission, resulting in extensive GBS colonization of the infant. The prematurity with low birthweight may also contribute to the development of GBS disease via immature immune system and mucosal barrier. Neonates have physiologic immunodeficiency of immaturity in early infancy, which is more severe when associated with prematurity and low birthweight for the following reasons: in premature neonates compared with term newborns, decreased total immunoglobulins G and M correlated with gestational age and birthweight due to low levels of maternal antibody that can pass transplacentally in late pregnancy and deficiency in endogenous immunoglobulin production^[22–24]^; slower postnatal maturation of the immunoglobulin G heavy chain repertoire^[25]^; reduced complement serum levels and activity.^[26]^ Previous experimental studies documented that C3 or C4 deficiency is associated with increased susceptibility to GBS infection^[27]^ and C3 deficiency is associated with marked impairment of GBS-induced tumor necrosis factor-α (TNF-α) release correlated with fatal infection.^[28]^ These may elucidate our observation of GBS sepsis with recurrence and relatively mild clinical presentation in the current case showing lower immunoglobulin and complement levels and decreased whole complement activity in the early neonatal period.

In this case, the likely source of recurrence may be subclinical mucosal persistence of GBS, evidenced by the nasal culture result 7 days after completion of the first apparently successful antimicrobial therapy. Despite lack of studies for genotypic relatedness of the isolates, recrudescence due to the original stain causing the early-onset sepsis may also be suggested, considering the relatively short 13-day interval between the episodes comparable to previous data (mean, 9.4 days; range, 4–19 days),^[7]^ similar antimicrobial profiles from all the isolates, and hospitalization period with no breastfeeding and direct maternal contacts, no known concurrent cases, and incubator care with strict hygiene policy for environmental infection control.

The progressive asymmetric mild to moderate ventriculomegaly and grade II PVL with cystic degeneration without intraventricular hemorrhage in our infant may represent WMI that may be linked to the 2 episodes of GBS sepsis with other previously defined relevant predictors including prematurity, maternal chorioamnionitis, and PROM.^[18,20,29,30]^ Previous human and experimental studies have been demonstrated the association of in utero and neonatal infection/inflammation with WMI and ventriculomegaly.^[13,31,32]^ In premature infants, ventriculomegaly may be a marker of WMI with better interrater reliability in assessment^[32]^ and may reflect neonatal diffuse noncystic WMI below resolution of presently available neuroimaging modalities, separate from concurrent small focal necrotic WMI.^[12]^ Leviton et al^[31]^ found that extremely preterm infant's placenta harboring low-virulence microorganisms was linked to echolucent WMI and ventriculomegaly, with the heightened ventriculomegaly risk when histologic chorioamnionitis was combined. Pappas et al^[32]^ reported that nonhemorrhagic ventriculomegaly was related to neonatal morbidities commonly associated with systemic inflammation. WMI has been documented to be associated with postnatal culture-proven infection including multiple infections, most commonly coagulase-negative *Staphylococcus* sepsis,^[14–17]^ however, only one case of GBS sepsis with WMI is found in 1 study.^[15]^

It has been suggested that bacteremia without microbial CNS invasion may induce brain injury. Bacteremia/sepsis itself may contribute to the development of brain insults without neuroinvasion by viable bacteria via the following mechanisms with published evidence: intravascular and brain endothelial cell *Toll*-like receptors (TLRs) triggering releases of proinflammatory cytokines/chemokines that cause increased permeability of the blood-brain barrier and myelin and myelin-producing cell damage after entry into the CNS, disseminated intravascular coagulopathy and alterations in cardiovascular performance such as hypotension and septic shock leading to decreased perfusion of the periventricular end-arterial zone, and increased susceptibility of the CNS to subsequent insults.^[13,31,33]^ Regarding the contribution of GBS bacteremia without bacterial CNS invasion to WMI, hypothetical mechanisms include pathways associated with neuronal damage after blood-brain barrier transport of intravascular cytokines and/or bacteria-derived components^[13]^: blood monocyte TLRs/myeloid differentiation antigen 88 (MyD88)-mediated release of TNF-α,^[34]^ which has been considered a myelinotoxic mediator as evidenced by a previous observation of in situ high expression of TNF-α in neonatal brains of 19 PVL cases,^[35]^ and cell wall preparations and secreted factors of GBS, which can induce neurodegeneration via microglial TLR2/MyD88-dependent activation.^[36]^ In the literature 4 preterm cases of PVL associated with neonatal GBS sepsis without meningitis from 2 reports are found.^[37,38]^ Unlike our case, these 4 cases were linked to early-onset GBS septic shock; of these 1 had no CSF study and 3 had GBS pneumonia with CNS presentations including convulsion or intracranial hemorrhage.^[37,38]^ The postnatal progressive WMI in our infant may be an example of WMI associated with GBS bacteremia without CNS entry by viable GBS, without symptoms and signs of meningitis, intraventricular hemorrhage, hypocarbia, coagulopathy, and hemodynamic derangement prompting volume and inotropic support.

## 5 Conclusion

This case shows that in premature infants, intrauterine GBS infection with no interventions may lead to extensive and persistent GBS colonization, early-onset and recurrent GBS disease, and WMI. Postnatal as well as intrauterine infection/inflammation controls with maternal prophylaxis may be pivotal for prevention and limiting the magnitude of neurologic injury.

## Acknowledgments

The authors thank the patient and her family for providing informed consent for the publication of this case report and accompanying images.

## Author contributions

**Approval of final manuscript:** Cheong-Jun Moon, Tae Hee Kwon, Kyung Sang Lee, Hyun-Seung Lee.

**Conceptualization:** Hyun-Seung Lee.

**Data curation:** Cheong-Jun Moon, Tae Hee Kwon, Kyung Sang Lee, Hyun-Seung Lee.

**Investigation:** Tae Hee Kwon, Kyung Sang Lee, Hyun-Seung Lee.

**Writing – original draft:** Cheong-Jun Moon.

**Writing – review & editing:** Hyun-Seung Lee.
